# Insecticidal Activities Against *Odontotermes formosanus* and *Plutella xylostella* and Corresponding Constituents of Tung Meal from *Vernicia fordii*

**DOI:** 10.3390/insects12050425

**Published:** 2021-05-10

**Authors:** Hui Zhang, Guilin Chen, Shiyou Lü, Lin Zhang, Mingquan Guo

**Affiliations:** 1 CAS Key Laboratory of Plant Germplasm Enhancement and Specialty Agriculture, Wuhan Botanical Garden, Chinese Academy of Sciences, Wuhan 430074, China; zhanghui183@mails.ucas.ac.cn (H.Z.); glchen@wbgcas.cn (G.C.); 2 Institute of Geochemistry, University of Chinese Academy of Sciences, Beijing 100049, China; 3 Sino-Africa Joint Research Center, Chinese Academy of Sciences, Wuhan 430074, China; 4 Innovation Academy for Drug Discovery and Development, Chinese Academy of Sciences, Shanghai 201203, China; 5 State Key Laboratory of Biocatalysis and Enzyme Engineering, School of Life Sciences, Hubei University, Wuhan 434200, China; shiyoulu@hubu.edu.cn; 6 Key Laboratory of Cultivation and Protection for Non-Wood Forest Trees, Ministry of Education, Central South University of Forestry and Technology, Changsha 410004, China; triwoodtim918@126.com

**Keywords:** euphorbiaceae, insecticidal activities, plant extracts, botanical pesticide, biological control

## Abstract

**Simple Summary:**

*Odontotermes formosanus* (Shiraki) (*O. formosanus*) and *Plutella xylostella* (Linnaeus) (*P. xylostella*) are common industrial and agricultural pests with a wide distribution range, multiple host species, few natural predators, and rapid reproduction. The control of these pests relies heavily on synthetic chemical pesticides; however, environmental pollution and the rise of insect resistance induced by the excessive use of synthetic chemical pesticides pose serious challenges. The present study was designed to systematically evaluate the insecticidal activities of different extracts of tung meal against the above two pests and to reveal the chemical constituents of the main active parts. The result confirmed the remarkable insecticidal activity of tung meal and provided a theoretical basis for its development as a potential plant insecticide.

**Abstract:**

The environmental pollution, pesticide resistance, and other associated problems caused by traditional chemical pesticides with limited modes of action make it urgent to seek alternative environmentally-friendly pesticides from natural products. Tung meal, the byproduct of the detoxified *Vernicia fordii* (Hemsl.) seed, has been commonly used as an agricultural fertilizer and as a pesticide. However, its active insecticidal extracts and ingredients remain elusive. In the present study, the contact toxicities of tung meal extracts against the agricultural and forest pests like *O. formosanus* and *P. xylostella* were examined. Our results showed that ethyl acetate and petroleum ether extracts showed the strongest toxicity against *O. formosanus* and *P. xylostella*, respectively. In order to further explore the chemical profiles of the ethyl acetate and petroleum ether extracts, UPLC-Q/TOF-MS and GC-MS analyses have been performed, and 20 and 29 compounds were identified from EA and PE extracts, respectively. The present study, for the first time, verified the noteworthy insecticidal activities on the aforementioned agricultural and forest pesticides and revealed the potential active parts and chemical composition, which are conducive to further exploiting the potential of tung meal as a natural plant-derived insecticide for biological control of agricultural and forest pests.

## 1. Introduction

*Vernicia fordii* (Hemsl.) Airy Shaw, a subtropical perennial industrial crop, is widely distributed in the east of Asia. It originated from China and is also known as tung oil tree. Phytochemical investigations on *V. fordii* have revealed the presence of fatty acids, lignans, terpenes, polyphenols, flavonoids, etc. [1]. As an industrial manufacture material, tung oil is often used for paints and coatings, and has also been developed as a renewable bioenergy source with great potential [2]. In particular, tung meal, the by-product of the de-oiling of tung seeds, is commonly used as fertilizer in agriculture due to its rich protein and nutrient content [3]. On the one hand, there are some limitations of tung meal in agriculture due to its toxic ingredients [4,5]; on the other hand, it has been revealed that the extracts of tung meal exhibited excellent insecticidal effects against some agricultural pests, like *Enchytraeus bulbosu*. However, there are few systematic studies focusing on its insecticidal activities and components [6,7]. To this end, it is of great importance to identify the effective insecticidal potential and the main components of tung meal for its further development in agriculture as natural insecticides.

*Odontotermes formosanus* (*O. formosanus*) belonging to Isoptera, also known as *termitidae*, is characterized by fast reproduction speed, wide distribution range, and high destructive power. This insect mainly harms gardens, trees, buildings, and dams, thus posing a serious threat to the wildlife, human health, and world forestry economy [8]. Statistically, more than $40 billion losses are caused directly or indirectly by termites annually, and *termitidae* has become one of the five major pests in the world [9]. *Plutella xylostella* (*P. xylostella*) is a widespread omnivorous insect belonging to the Lepidoptera and Plutellidae [10]. It has been one of the most harmful pests to cruciferous vegetables in many countries worldwide due to its numerous host species, few natural predators, pesticide resistance, and rapid reproduction rate, and there remain great challenges to be confronted in the control of *P. xylostella* [11,12].

At present, the chemical control of these pests is still primarily based on the use of synthetic chemical pesticides, which have been sustained over many decades. However, diverse challenges are faced due to the excessive usage of traditional synthetic pesticides, such as pesticide residues, susceptibility to pesticide resistance, environmental and human health problems [13]. For example, current highly toxic drugs to control termites mainly include imidacloprid, cypermethrin, permethrin, fipronil, etc. In addition, *P. xylostella* has developed resistance to a variety of insecticidal chemicals, including pyrethroids, carbamate, organic fluidics, organochlorines, abamectin, polyfungicides, and even *Bacillus thuringiensis*, which makes it increasingly difficult and costly to control. With the banning of highly toxic insecticides and the increasing resistance to some commonly used insecticides, finding environment-friendly biological pesticides is now becoming particularly urgent [14]. 

The ecological and human health problems caused by chemical pesticides have been prompting extensive searches for environment-friendly biological pesticides [15]. Medicinal plant resources are abundant and easy to obtain. Extracting high-efficiency, biodegradable, and low-toxicity or non-toxic active ingredients from medicinal plants for pest management can not only reduce environmental pollution, but also be helpful in reducing pesticide resistance [16]. In addition, compared with traditional insecticides, plant-derived insecticides also have some other advantages like leaving no residue, various types and modes of action, etc., which play a positive role in solving the problems caused by chemical pesticides [17,18]. Considering the necessity and urgency for the development of environment-friendly bio-insecticides, this research aimed to investigate the insecticidal activities of tung meal against the above two pests and reveal the chemical constituents of the extracts with the strongest toxicity [5]. The results provide further practical guidance for the agricultural application and development of tung meal as both natural plant-based insecticides and organic fertilizers.

## 2. Materials and Methods

### 2.1. Collection and Rearing of Insects 

The *O. formosanus* was collected from Shizishan, Hongshan district, Wuhan city, Hubei province. They were kept in a box (30 × 15 × 15 cm) in a dark environment of 25 ± 1 °C and 70 ± 5% RH (relative humidity). The larvae of *P. xylostella* (Linnaeus) were purchased from Henan Jiyuan Baiyun Industry Co., and grown in a culture chamber at 27 ± 1 °C and 60 ± 10% RH; the illumination period was 14 L:10 D.

### 2.2. Acquisition of Plant Materials and Extracts

Tung meal of *V. fordii * Hemsl. was collected from Qingping in June 2018, Hunan province. The specimen was kindly authenticated by plant taxonomist professor Guangwan Hu and deposited at Wuhan Botanical Garden, Chinese Academy of Sciences. Firstly, 3 kg of dry tung meal powder was soaked in 15 L of 95% ethanol for 11 h. Then, ultrasonic extraction was performed in triplicate at 200 W and 40 KHz for 30 min, and the extracts were concentrated into crude syrup residues using a rotary evaporator. Later, the crude extracts (CE) were dispersed in water and partitioned with petroleum ether (PE), dichloromethane (DCM), ethyl acetate (EA), and n-butanol (n-Bu) successively, the remaining water layer was the aqueous extraction part (H_2_O). Finally, the corresponding dry extracts were obtained after freeze-drying. For the following LC-MS analysis, the EA extracts were re-dissolved with methanol and then filtered with a 0.22 μm filter membrane before injection. As for the following GC-MS analysis, the PE extracts were re-dissolved with n-hexane, and then filtered through a 0.22 μm filter membrane before injection. 

### 2.3. Insecticidal Bioassay

#### 2.3.1. Insecticidal Activity on Forest Pest *O*. *Formosanus*

The termite-resistance activity of tung meal against *O. formosanus* was determined by contact with filter paper containing samples with different concentrations. Briefly, appropriate amounts of different extracts (1 mL) were evenly dropped on the qualitative filter paper (9 cm in diameter) and spread in a 9 cm diameter glass petri dish. Twenty worker termites of about the same size were put into each plate under 25 ± 1 °C and 70 ± 5% RH in the dark. Deaths of worker termites were recorded and eliminated every 24 h, and the observation was continued until all termites died. The test samples were diluted with dimethyl sulfoxide (DMSO) into the initial concentration of 10 mg/mL, and then diluted with distilled water to different concentrations (10, 5, 2.5, 1.25, 0.625 mg/mL). Distilled water and DMSO solution with the same concentration were used as controls. Abbott formula (Abbott, 1925) was used for survival rate correction, and the adjusted survival rate of 6 extracts after 24, 48, and 72 h were calculated, respectively.

#### 2.3.2. Insecticidal Activity on Agricultural Pest *P. xylostella*

The insecticidal activity of tung meal against *P. xylostella* was measured by feeding a diet containing extracts with different concentrations. Twenty-four *P. xylostella* in the second instar larvae were used as test samples; the larvae of *P. xylostella* were fed with different formulated feed (1 mg sample/1 g normal feed) after starvation treatment for 6 h, and the deaths were counted and recorded every 24 h. The formulated feed was prepared as follows: tung meal extracts (0.005 g) were firstly dissolved in the solvents of 100 μL DMSO and 150 μL H_2_O; then the above solutions were mixed and stirred with 10 g of normal feed; finally, the prepared formulated feed was air-dried and stored at 4 °C. Distilled water and DMSO solution with the same concentration were used as controls. The analytical methods conducted were as the same as described in Section 2.3.1.

### 2.4. Chemical Composition Analysis

#### 2.4.1. UPLC-Q/TOF-MS Analysis of EA Extracts

The chemical constituents of the EA extracts from tung meal were analyzed using an Agilent 1290 LC coupled with an Agilent 6530 accurate mass time-of-flight mass spectrometer (Agilent, Santa Clara, CA, USA) equipped with an Acquity UPLC-BEH-C18 column (2.1 × 50 mm, 1.7 µm; Waters, Milford, MA, USA). The Q/TOF-MS instrument was equipped with a dual ESI source in the negative ionization mode. The mobile phase consisted of mobile phase A (0.1% formic acid in ultrapure water) and mobile phase B (acetonitrile). The LC gradient elution was as follows: 5–27% B, 0–29 min; 27–32% B, 29–35 min; 32–95% B, 35–45 min; 95% B, 45–50 min. The injection volume was 10 µL, and the flow rate was 0.2 mL/min. The MS was performed under the following conditions: Mass Hunter workstation (Agilent) was used to obtain profile data with a mass range of *m/z* 100–1100 at a rate of 1 spectra/s. The temperature and the gas flow rate of drying gas were set at 350 °C and 8 L/min, respectively. The nebulizer pressure was set at 35 psi. The capillary voltage (Vcap) and fragment voltage were set at 3500 V and 175 V. The fixed collision energies were 10, 20, and 40 V, and reference masses of *m/z* 112.9855 and 1033.9881 were continuously introduced for accurate mass calibration. Compounds in the extracts of tung meal were tentatively identified by comparing the parent ion, retention time, and mass fragments with the correlated references and database.

#### 2.4.2. GC-MS Analysis of PE Extracts

The identification of compounds in PE extracts was carried out in accord with the previous methods with slight modification [19,20]. Identification was performed by the gas chromatographic system (Agilent 7890A, Agilent, Santa Clara, CA., USA) combined with MS system (Agilent 7000C, Agilent, Santa Clara, CA., USA) equipped with an HP-5 capillary column. Gas chromatography is mainly used for the separation of volatiles. The carrier gas is helium with a flow rate of 1 mL/min. The GC column temperature program was initially set at 100 °C for 8 min and gradually increased to 220 °C at 5 °C/min, then at 2 °C/min and gradually increased to 275 °C, and finally at 5 °C/min and gradually increased to 310 °C and maintained for 10 min. The electron ionization (EI) system was used to obtain the mass spectrum with a mass range of 25–500 Da; the ionization energy is 70 eV in GC-MS detection. Identification of chemical composition was performed by using the NIST 11 Mass Spectral Library. The relative percentages of each compound were calculated by computerized integrator using total ion chromatography.

### 2.5. Statistical Analysis

All statistical analysis was performed employing SPSS Statistics version 24.0 software (IBM Corporation, Armonk, NY., USA), and experimental results were expressed as mean value (obtained by three replicates) ± standard deviation. Statistical differences between groups were analyzed by one-way analysis of variance (ANOVA) combined with Duncan’s test (*p* < 0.05). 

## 3. Results and Discussion

### 3.1. Insecticidal Activity of Tung Meal on Forest Pest O. Formosanus

The insecticidal activity of tung meal against *O. formosanus* was represented by the survival rate after 72 h of treatment. It can be seen from Table S1 and Figure S1 that the insecticidal activity of tung meal varied with the types of extracts, exposure time, and concentration.

Within the concentration range of 2.5 to 10 mg/mL, different tung meal extracts showed diverse degrees of insecticidal activities against *O. formosanus,* among which the EA extracts were superior to the others. The detailed results are shown in Table S1. When the concentration was 10 mg/mL, the above 6 different tung meal extracts displayed comparable insecticidal activities against *O. formosanus*, with the equivalent survival rate of 0% after treatment for 72 h; when the concentration was cut to 5 mg/mL, the survival rates of the EA and PE extracts remained at 0%, while the DCM, CE, n-Bu, and H_2_O extracts were 1.25%, 3.75%, 3.75%, and 13.75%, respectively; when the concentration came to 2.5 mg/mL, the survival rate of the EA extracts also remained the lowest at 23.75%, and the other groups were 41.25% (DCM), 47.5% (CE), 56.25% (n-Bu), 25% (H_2_O), and 85% (PE). All extracts, except the PE extracts, were significantly lower (*p* < 0.05) than that of distilled water (97.50%) and the DMSO control group (97.50%) after 72 h of treatment with 2.5 mg/mL of samples. When the concentration was lower than 1.25 mg/mL, the survival rate of different extracts and fractions was more than 95%, indicating that the effective concentration of tung meal extracts and fractions against *O. formosanus* should be higher than 1.25 mg/mL.

From these results, it can be concluded that tung meal has remarkable toxic activity against *O. formosanus*. Meanwhile, the EA extracts were the most promising as an insecticide, and the active components are mainly some compounds with moderate polarity. Interestingly, our present results were similar to the previous study of Sillma [21], in which they evaluated the insecticidal effects of different extracts from *Jatropha curcas*, another common plant of Euphorbiaceae, on two insect larvae of *Bactrocera zonata* and *Bactrocera cucurbitae*. They found that *J. curcas* exerted significant insecticidal effects on the two larvae, among which EA extracts were the most active part.

### 3.2. Insecticidal Activity of Tung Meal on Agricultural Pest P. xylostella

The influence of different extracts obtained from *V. fordii* on the survival rate of *P. xylostella* is shown in Table 1. Among all the extracts, PE extracts showed the most significant toxic activity against *P. xylostella* with a survival rate of 58.33% after adding 1 mg of the active ingredients into 1 g of normal feed for 72 h, whereas the n-Bu extracts (60.42%) displayed the second-highest response. EA and CE extracts led to similar survival rates of 75.00%. The survival rate for the above four extracts was significantly lower than that of DMSO and water control (*P* < 0.05); the survival rates for the other two extracts were 79.17% (H_2_O) and 85.42% (DCM). 

In this study, PE extracts of tung meal presented the highest insecticidal effect on *P. xylostella*, indicating that the components with toxic activity to *P. xylostella* were mainly low-polarity compounds, and the PE extracts of tung meal were more suitable to be further developed as plant-derived insecticides against *P. xylostella*. Therefore, further analysis of the chemical components of PE extracts was later conducted to reveal the potentially responsible compounds that might play a role in the insecticidal activity of tung meal and to explore the potential of tung meal as a botanical insecticide.

### 3.3. Characterization of Chemical Components in EA Extracts by UPLC-Q/TOF-MS

In natural ecosystems, a wide variety of secondary compounds are involved in plant defenses against insects, such as insect repellents and toxins. Our study revealed the insecticidal activity of tung meal extracts against *O. formosanus and P. xylostella*. However, the complex chemical components in the tung meal extracts with very low content make them very difficult to be identified using the traditional methods often involving tedious isolation, purification, and NMR identification which greatly limit the exploration of their chemical components. UPLC-Q/TOF-MS has become an effective method for the identification of complex natural products combining its rapid and efficient separation ability of UPLC with the highly sensitive and accurate detection capacity of TOF-MS. In this study, UPLC-Q/TOF-MS was used to rapidly identify the chemical constituents in the EA extracts of tung meal which exhibited the best activity against *O. formosanus* as shown in Table 1.

**Figure 1:**
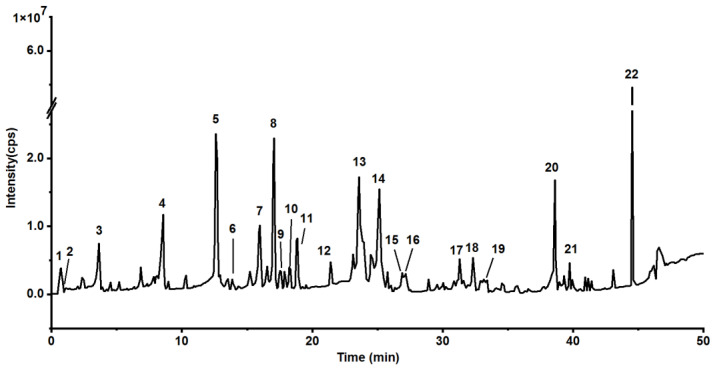
The base peak chromatogram (BPC) of EA extracts from tung meal.

A total of 20 compounds were tentatively identified from the EA extracts of tung meal by comparing their retention times and MS fragments with the data in the references. Each compound was numbered according to the retention time of the base peak chromatography (BPC) (Figure 1). The retention times, molecular formula and weight, and MS fragments of all compounds are listed in Table 2.

#### 3.3.1. Lignans

**Figure 2:**
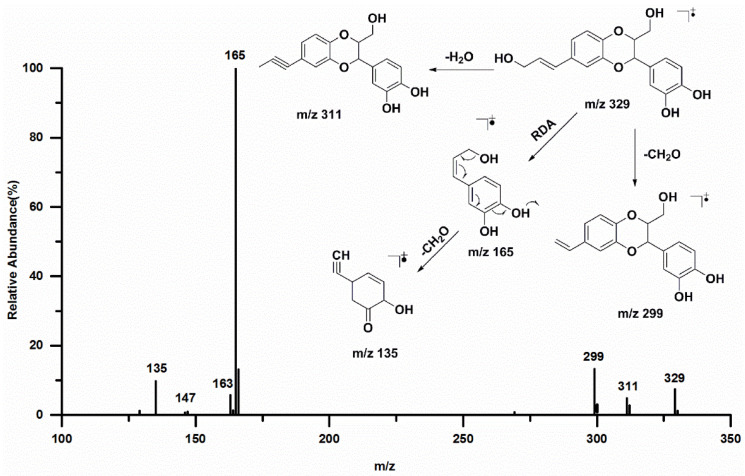
The fragmentation pathways based on the MS/MS spectra of isoamericanol A.

Lignans are common components of Euphorbiaceae plants. As illustrated in Table 2, fourteen peaks (peaks 1, 5, 6, 8, 10, 11, 12, 13, 14, 15, 16, 17, 18, and 19) were tentatively identified and classified as lignans. Compounds 8 and 11 exhibited a similar parent molecular ion [2M-H]^−^ at *m/z* 659 (t_R_ 17.06 min and 18.86 min) and MS fragments. The deprotonated molecular ion [M-H]^−^ was shown at *m/z* 329. A characteristic ion can be seen at *m/z* 165 due to the Retro Diels–Alder (RDA) cleavage, and the fragment ions at *m/z* 147 and *m/z* 137 could be attributed to the continuous loss of H_2_O and CO. The fragment ion at *m/z* 163 was due to the cleavage of 1,4-dioxane, and the additional loss of CH_2_O yielded fragments at *m/z* 135. Figure 2 shows the MS spectrum and the corresponding proposed fragment pathways. Hence, peaks 8 and 11 were tentatively assigned as americanol A and isoamericanol A, which is in good agreement with the previous report [23].

Compounds 1 and 5 were detected at t_R_ 0.68 and 12.59 min with similar deprotonated molecular ion [M-H]^−^ of *m/z* 329 and MS spectral data, which indicated that they were the isomers. Two distinguished fragments at *m/z* 329 and *m/z* 137 were generated by deprotonation or the cleavage of the furan ring. The fragment ions at *m/z* 299 and *m/z* 269 indicated the continuous release of the CH_2_O group from [M-H]^−^ and [M-H-CH_2_O]^−^. A characteristic ion can be seen at *m/z* 165 due to the RDA cleavage. The MS data were in agreement with those previously reported, and compounds 1 and 5 were identified as (+)-7-epi-sesamin-dicatechol and (±)-3,3′-bisdemethylpinoresinol, respectively [22,23]. 

Compound 12 displayed a deprotonated molecular ion [M-H]^−^ of *m/z* 327 at t_R_ 21.39 min, which was 2 Da (2 hydrogens) less than americanol A and isoamericanol A. The fragment at *m/z* 297 was attributed to the neutral loss of CH_2_O. Typical fragment ions of compound 12 were obtained at *m/z* 165, *m/z* 163, *m/z* 147, and *m/z* 135, which were similar to americanol A and isoamericanol A. Compound 12 was identified as isoamericanin A, where one hydroxymethyl group in isoamericanol A was oxidized to an aldehyde group, in comparison with their spectral features. This compound had also been found in the seed testa of *Vernicia fordii* [23]. 

There are six neolignan isomers including peaks 6, 10, 13, 14, 15, and 16, which were detected at t_R_ 13.84, 18.23, 23.59, 25.14, 26.88, and 27.17 min, respectively. The deprotonated molecular ion [M-H]^-^ was observed at *m/z* 493. The fragment ions of *m/z* 475 and *m/z* 463 indicated the easier loss of the H_2_O and CH_2_O groups. The additional loss of C_9_H_8_O_3_ group yielded a fragment at *m/z* 299. The major fragment ions of *m/z* 329 and *m/z* 163 resulted from the cleavage of 1,4-dioxane. The fragment ion of *m/z* 165 was similar to americanol A and isoamericanol A. By comparing the fragmentation pathways with those of the references, compounds 6, 10, 13, 14, 15, and 16 could be tentatively identified as isoamericanol B1, isoamericanol B2, isoamericanol C1, isoamericanol C2, princepin, and isoprincepin [24], which was in accord with the findings of Chen et al [23]. Those compounds were also named sesquineolignans because they were structurally formed by the oxidative polymerization of three phenylpropyl. In addition, all of these chemical components had also been found in the seeds of *Joannesia princeps* Vellozo, another plant of the Euphorbiaceae [25]. 

Three dilignant isomers, compounds 17, 18, and 19, were detected at t_R_ 31.28, 32.31, and 33.40 min with the same deprotonated molecular ion [M-H]^−^ of *m/z* 657 and MS spectral data. The characteristic fragment ions of *m/z* 493 and *m/z* 163 resulted from the cleavage of 1,4-dioxane. Another major characteristic fragment ion of *m/z* 165 was also observed. Furthermore, fragment ions of *m/z* 493 and other fragment ions were similar to those of (iso) princepin. Those three compounds were tentatively identified as isodiverniciasin A, diverniciasin C, and diverniciasin B, which have been detected in the seed testa of *V. fordii* [23].

#### 3.3.2. Fatty Acids

Compound 7 exhibited the deprotonated molecular ion at *m/z* 187 [M-H]^−^ at t_R_ 15.98 min. The fragment ions at *m/z* 169 and *m/z* 143 were obtained by the successive loss of H_2_O and CO_2_ groups, respectively. The major fragment ion at *m/z* 125 was obtained by the further loss of C_2_H_4_O from the [M-H-H_2_O]^−^. According to the literature [26] and the discussion mentioned above, compound 7 could be identified as azelaic acid. Compound 20, which appeared at the t_R_ 38.60 min, exhibited a parent ion at *m/z* 309. The fragment ions at *m/z* 291 and *m/z* 185 could be attributed to the loss of H_2_O or C_9_H_16_, and fragment ion at *m/z* 209 was generated by the successive loss of C_6_H_10_. According to the Mass Bank Database and the discussion mentioned, compound 20 was tentatively assigned as (9S,10E,12Z,15Z)-9-hydroperoxyoctadeca-10,12,15-trienoic acid.

Compound 21 was observed at *m/z* 307 (t_R_ 39.73 min). The abundant fragment ions at *m/z* 289 and *m/z* 185 could be attributed to the loss of H_2_O or C_8_H_10_O. The additional loss of CH_4_O_2_ group yielded fragment ion at *m/z* 137. These mass spectra matched segments of the Mass Bank Database; therefore compound 21 was preliminarily designated as corchorifatty acid D.

#### 3.3.3. Others

Compound 2, which appeared at the retention time of 0.82 min, revealed a [M+FA-H]^-^ ion at *m/z* 387. It yielded the productions at *m/z* 179 and *m/z* 161 in the MS/MS by the loss of glucose (180 Da) or glucose residue (162 Da). Furthermore, a smaller fragment resulting from the cleavage of the hexose ring was observed at *m/z* 89 (C_3_H_5_O_3_). According to the literature [27] and the discussion mentioned, compound 2 could be identified as trehalose. Compound 3 presented the deprotonated molecular ion *m/z* 137 [M-H]^-^ at t_R_ 3.65 min. The distinguished fragment ion of *m/z* 119 indicated the loss of H_2_O, and another major fragment ion *m/z* 108 was obtained by the loss of CO. Considering that the above fragments were the same as in the previous report [28], compound 3 was tentatively identified as 2,4-dihydroxybenzaldehyde, which had been detected in the seed testa of *V. fordii* [23].

The electrospray mass spectrum of compound 9 exhibited a parent ion of *m/z* 663 [2M-H]^-^ at t_R_ 17.53 min. Two characteristic fragments at *m/z* 331 and *m/z* 313 were generated by deprotonation and the loss of H_2_O. The fragment [M-H-OCH_3_]^−^ was found at *m/z* 301. The ions at *m/z* 165 and *m/z* 147 were the typical fragments of RDA cleavage and their dehydrated fragments. These fragments were similar to the mass spectrometry fragments in the Human Metabolome Database (HMDB), and compound 9 was preliminarily identified as 5,7,4′-tri-*O*-methylcatechin.

**Figure 3:**
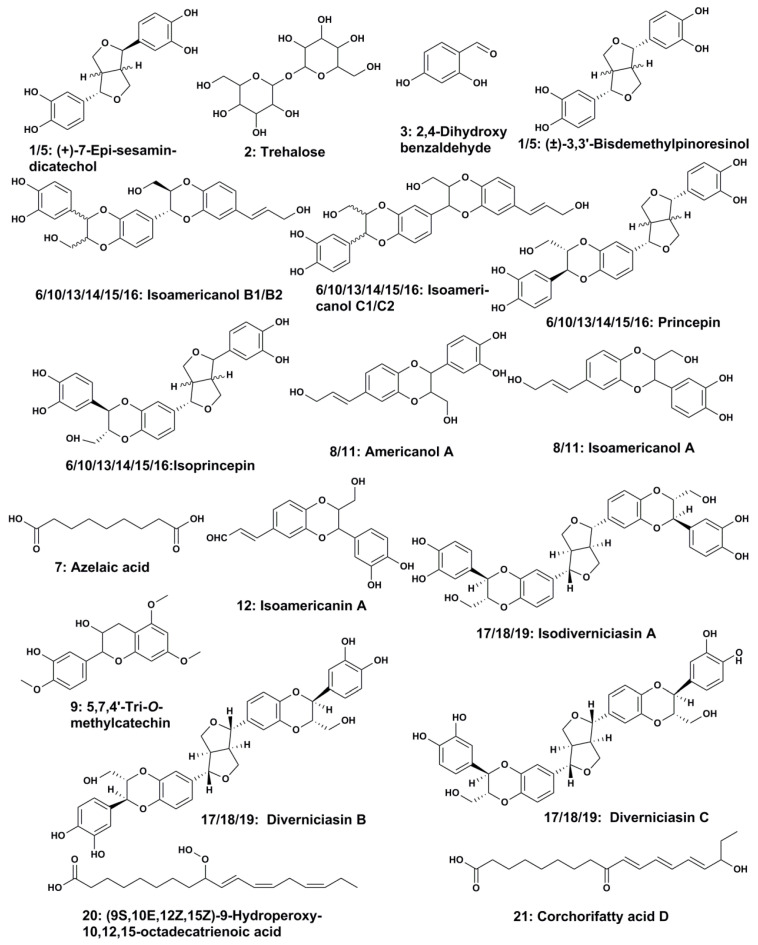
The structures of compounds identified from EA extracts of tung meal by UPLC-Q/TOF-MS.

According to the structures of these compounds shown in Figure 3, these components can be structurally characterized as lignans, fatty acids, flavonoids, disaccharides, and dihydroxybenzaldehyde. According to their basic carbon frames, those lignans can be divided into three categories, including monolignan (compounds 1, 5, 8, 11, and 12), sesquineolignan (compounds 6, 10, 13, 14, 15, and 16), and dineolignans (compounds 17, 18 and 19). By integrating the chromatogram, it was found that the contents of isoamericanol B1 and its related isomers (isoamericanol B2, isoamericanol C1, isoamericanol C2, isoprincepin, and isoprincepin) were the highest in the EA extracts of tung meal, accounting for 24.01% of the total, followed by (+)-7-epi-sesamin-dicatechol and its related isomers (±)-3,3′-bisdemethylpinoresinol, which also accounted for a high proportion of 13.58%; besides, americanol A and its related isomer isoamericanol A also accounted for a high proportion of 10.14%. 

Previous studies revealed that trehalose can remarkably reduce the mean day survival rate of sweet potato whitefly *Bemisia tabaci* (Gennadius) [29], and trehalose/mannose/maltose specific lectins exhibited promising insecticidal activities against stored product pests [30]. Azelaic acid was also detected at the active sites of a variety of plants with remarkable insecticidal activities [31]. The insecticidal activity of lignans has been rarely studied or not yet discovered. Some lignans, such as (±)-3,3′-bisdemethylpinoresinol, americanol A, americanin A, and isoprincepin, have been found in the fruit of *Morinda citrifolia* [32], and the fruit extracts of *Morinda citrifolia* have been reported to exert significant insecticidal activities against a variety of agricultural pests and have been used as an effective insecticide in the Philippines and Hawaii [33]. Princepin and isoprincepin are uncommon in natural products and their insecticidal activities have been poorly studied, but their structure-related compound haedoxan A has been reported to have prominent insecticidal activity [34,35]. Furthermore, *J. curcas* has been reported to have insecticidal activities on houseflies and insect pests of stored products [36,37]. In this study, we detected some major components in tung meal, such as isoamericanol A, americanol A, (+)-7-Epi-sesamin-dicatechol, (±)-3,3′-Bisdemethylpinoresinol, and isoprincepin, which are also the main components of *J. curcas* [38]. In conclusion, the insecticidal activity of tung meal could be attributed to the presence of those components; however, the insecticidal activities and their mechanisms based on these potential components need to be further verified.

### 3.4. Identification of Volatile Constituents in PE Extracts by GC-MS

**Figure 4:**
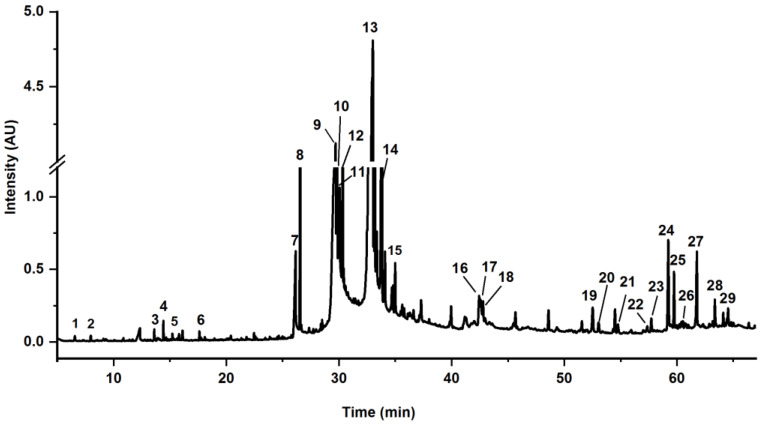
The GC-MS chromatogram of PE extracts from tung meal.

The components in PE extracts were tentatively identified by comparing the MS fragments with the NIST database. Based on the literature, a compound whose similarity scores above 90% was taken into account [39]. The gas chromatograms of PE extracts and the corresponding compounds tentatively identified in the extracts, along with the retention times and percentages are shown in Figure 4 and Table 3, respectively. According to the retention times peaked in the GC-MS analysis, a total of twenty-nine compounds, accounting for 87.26%, were identified, which mainly consisted of unsaturated fatty acids and their ethyl ester, phytosterols, etc. 

Among those compounds, the highest peak appeared at t_R_ 33.00 min, accounting for 38.44% of the total content, which was estimated as ethyl α-linolenate or ethyl γ-linolenate according to the MS data. The third highest peak appeared in t_R_ 33.82 min, accounting for 8.93% of the total content, and was tentatively identified as the isomer of the highest peak due to the same MS/MS fragments. The second highest peak appeared in t_R_ 29.69 min, accounting for 14.36% of the total content, and was tentatively identified as ethyl linolate. In addition, ethyl oleate (6.34%), ethyl palmitate (3.82%), ethyl stearate (3.09%), stearic acid (2.38%), palmitic acid (2.09%), and *β*-sitosterol (1.58%) were also tentatively identified in PE extracts. These results were consistent with previous studies [40,41].

Fatty acids and their ethyl ester, and *β*-sitosterol may be the principal components exerting remarkable insecticidal activity against *P. xyloides*. For example, ethyl linolenate, ethyl oleate, ethyl palmitate, ethyl stearate, and ethyl linolate were found in *Eupatorium odoratum* extract, an insect repellent, by inhibiting oviposition of *P. xyloides* [42]. The volatile oils and crude extracts of *Zingiber officinale* have noteworthy insecticidal, repellent, and oviposition activities against many pests like *Callosobruchus chinensis*, *Sitophilus zeamais*, and *P. xylostella* [43]. In a similar study, five components with a content of more than 2% have also been detected in *Z. officinale* essential oil and oleoresins, including palmitic acid, ethyl palmitate, ethyl linolate, ethyl oleate, and stearic acid [44]. In addition, the ethanol extracts of *Cynanchi auriculati* showed superior insecticidal efficacy to common pesticides against *P. xylostella*, among which PE extracts was the highest, and the analysis of chemical composition showed that it was rich in *β*-sitosterol [45].

## 4. Conclusions

In the present study, different extracts from the deoiled tung meal of *V. fordii* were first revealed to exhibit diverse insecticidal activities against *O. formosanus* and *P. xylostella*, respectively. The corresponding active extracts and potential components, including lignans, fatty acids and their derivatives, and others, were further confirmed by UPLC-Q/TOF-MS and GC-MS, which provided strong support for alternative forest and agricultural pest control by tung meal. Our results suggest that tung meal extract or its derivatives may be used to control and manage the infestation of some pests like *O. formosanus* and *P. xylostella*, as well as to mitigate the damage caused by chemical pesticides currently in use.

## Figures and Tables

**Table 1:** Insecticidal activity of different tung meal extracts on *O. formosanus* and *P. xylostella*.

Extracts	Survival Rate	
	*O. formosanus*	*P. xylostella*
DMSO	97.50 ± 2.50 a	97.92 ± 2.08 a
Control	97.50 ± 4.33 a	95.83 ± 4.17 a
CE	47.5 ± 16.01 b	75.00 ± 8.33 bcd
PE	85 ± 8.66 a	58.33 ± 8.33 d
DCM	41.25 ± 20.73 bc	85.42 ± 2.08 ab
EA	23.75 ± 10.23 c	75.00 ± 4.17 bcd
n-Bu	56.25 ± 12.44 b	60.42 ± 6.25 cd
H_2_O	25 ± 9.35 c	79.17 ± 4.17 abc

The different letters (a–d) after the mean value represent significant differences at *p* < 0.05 level according to Duncan’s test.

**Table 2:** Compounds tentatively identified from EA extracts of tung meal by UPLC-Q/TOF-MS.

No.	t_R_^a^ (Min)	Components	Content ^b^ (%)	Formula	Measured Mass (*m/z*)	MS1 (*m/z*)	MS/MS (*m/z*)	Error (ppm)
1	0.68	(+)-7-Epi-sesamin-dicatechol/(±)-3,3′-Bisdemethylpinoresinol	1.42	C_18_H_18_O_6_	329.1025	329.1043	329, 299, 269, 165, 137, 109	5.47
2	0.82	Trehalose	0.46	C_12_H_22_O_11_	387.1139	387.1168	341, 179, 161, 143	7.49
3	3.65	2,4-dihydroxybenzaldehyde	2.84	C_7_H_6_O_3_	137.0239	137.0241	137, 119, 109, 108	1.46
4	8.55	Unknown	5.47	-	-	467.1955	467, 403, 343, 331, 313, 233, 203, 185	-
5	12.59	(+)-7-Epi-sesamin-dicatechol/(±)-3,3′-Bisdemethylpinoresinol	12.16	C_18_H_18_O_6_	329.1025	329.1048	329, 269, 189, 165, 137, 109	6.99
6	13.84	Isoamericanol B1 or related isomers (B2/C1/C2/(iso)princepin)	0.54	C_27_H_26_O_9_	493.1499	493.1513	493, 475, 463, 329, 327, 281, 165, 163	2.84
7	15.98	Azelaic acid	4.38	C_9_H_16_O_4_	187.0979	187.0987	187, 169, 143, 125	4.28
8	17.06	(iso)americanol A	7.51	C_18_H_18_O_6_	659.2129	659.2154	329, 311, 299, 165, 147, 135	3.79
9	17.53	5,7,4′-Tri-*O*-methylcatechin	1.13	C_18_H_20_O_6_	663.2442	663.2473	332, 331, 313, 301, 165, 147, 135	4.68
10	18.23	Isoamericanol B1 or related isomers (B2/C1/C2/(iso)princepin)	1.10	C_27_H_26_O_9_	493.1499	493.1534	493, 463, 329, 327, 299, 269, 165, 163, 137	7.10
11	18.86	(iso)americanol A	2.63	C_18_H_18_O_6_	659.2129	659.2176	329, 311, 299, 165, 147, 135	7.13
12	21.39	Isoamericanin A	0.93	C_18_H_16_O_6_	327.0869	327.0890	327, 297, 165, 163, 147, 135	6.42
13	23.59	Isoamericanol B1 or related isomers (B2/C1/C2/(iso)princepin)	11.87	C_27_H_26_O_9_	493.1499	493.1528	493, 475, 463, 329, 327, 165, 163, 137	5.88
14	25.14	Isoamericanol B1 or related isomers (B2/C1/C2/(iso)princepin)	8.67	C_27_H_26_O_9_	493.1499	493.1509	493, 463, 329, 327, 165, 163	2.03
15	26.88	Isoamericanol B1 or related isomers (B2/C1/C2/(iso)princepin)	0.69	C_27_H_26_O_9_	493.1499	493.1518	493, 329, 327, 165, 163	3.85
16	27.17	Isoamericanol B1 or related isomers (B2/C1/C2/(iso)princepin)	1.14	C_27_H_26_O_10_	493.1499	493.1512	493, 477, 465, 329, 327, 299, 165, 163, 147, 135	2.64
17	31.28	Isodiverniciasin A/diverniciasin B/diverniciasin C	2.70	C_36_H_34_O_13_	657.1972	657.2005	657, 493, 461, 329, 327, 165, 163	5.02
18	32.31	Isodiverniciasin A/diverniciasin B/diverniciasin C	2.25	C_36_H_34_O_13_	657.1972	657.2018	657, 493, 461, 329, 327, 165, 163	7.00
19	33.40	Isodiverniciasin A/diverniciasin B/diverniciasin C	1.28	C_36_H_34_O_13_	657.1972	657.1973	657, 493, 461, 329, 327, 166, 163	0.15
20	38.60	(9S,10E,12Z,15Z)-9-Hydroperoxyoctadeca-10,12,15-Trienoic acid	4.25	C_18_H_30_O_4_	309.2066	309.2093	309, 291, 225, 209, 185	8.74
21	39.73	Corchorifatty acid D	0.84	C_18_H_28_O_4_	307.1910	307.1930	307, 289, 265, 223, 185, 137	6.51
22	44.54	Unknown	10.25	_-_	339.2374	-	339, 183, 163, 147	-

^a^ Retention time according to the base peak chromatogram in Figure 1. ^b^ The relative content (%) was obtained by integrating the chromatogram peaks in Figure 1.

**Table 3:** Compounds estimated from PE extracts of tung meal by GC-MS.

No.	t_R_^a^ (min)	Content (%)	Components ^b^	Formula	SC ^c^
1	6.56	0.06	Octanoic acid	C_8_H_16_O_2_	93
2	7.97	0.04	2,4-Decadienal	C_10_H_16_O	91
3	13.61	0.11	Caryophyllene	C_15_H_24_	99
4	14.42	0.14	(-)-*α*-Gurjunene	C_15_H_24_	91
5	15.21	0.08	*β*-Copaene	C_15_H_24_	93
6	17.62	0.07	(-)-Spathulenol	C_15_H_24_O	90
7	26.15	2.09	Palmitic acid	C_16_H_32_O_2_	93
8	26.56	3.82	Ethyl palmitate	C_18_H_36_O_2_	99
9	29.69	14.36	Ethyl linolate	C_20_H_36_O_2_	99
10	29.83	6.34	Ethyl oleate	C_20_H_38_O_2_	97
11	30.06	2.38	Stearic acid	C_18_H_36_O_2_	99
12	30.33	3.09	Ethyl stearate	C_20_H_40_O_2_	99
13	33.00	38.44	Ethyl *α*-linolenateor Ethyl *γ*-linolenate	C_20_H_34_O_2_	98
14	33.82	8.93	Ethyl α-linolenate or Ethyl γ-linolenate	C_20_H_34_O_2_	98
15	34.99	0.7	2,2′-Methylenebis(4-methyl-6-tert-butylphenol)	C_23_H_32_O_2_	94
16	42.44	0.69	2-Linoleoylglycerol	C_21_H_38_O_4_	90
17	42.57	0.48	2-Monoolein	C_21_H_40_O_4_	97
18	42.78	0.31	Heptacosane	C_27_H_56_	91
19	52.52	0.58	*γ*-Tocopherol	C_28_H_48_O_2_	99
20	53.04	0.25	Stigmasta-3,5-diene	C_29_H_48_	95
21	54.75	0.18	Vitamin E	C_29_H_50_O_2_	99
22	57.36	0.14	Dotriacontane	C_32_H_6_O_6_	96
23	57.72	0.23	Stigmasterol	C_29_H_48_O	99
24	59.23	1.58	*β*-Sitosterol	C_29_H_50_O	92
25	59.74	0.73	Tritriacontane	C_33_H_68_	99
26	60.52	0.09	4,22-stigmastadiene-3-one	C_29_H_46_O	96
27	61.76	0.96	Stigmast-4-en-3-one	C_29_H_48_O	98
28	63.36	0.34	Pentatriacontane	C_35_H_72_	97
29	64.53	0.36	Stigmastane-3,6-dione	C_29_H_48_O_2_	91

^a^ Retention time according to the GC-MS chromatogram in Figure 4. ^b^ The relative content (%) was obtained by integrating the chromatogram peaks in Figure 4. ^c^ Molecular weight; ^d^ Similarity score obtained by matching NIST 11.

## Data Availability

All data analyzed in this study are included in this article and supplementary materials.

## Supplementary Materials

The following are available online at https://www.mdpi.com/article/10.3390/insects12050425/s1, Table S1: Insecticidal activity of different tung meal extracts on termite of *O. formosanus*, Figure S1: Insecticidal activity of different tung meal extracts on *O. formosanus* and *P. xylostella*. The different letters (a-c) after the mean value represent significant differences at *p* < 0.05 level according to Duncan Test.
