# Supplementary material for: Insecticidal Activities Against Odontotermes formosanus and Plutella xylostella and Corresponding Constituents of Tung Meal from Vernicia fordii
Source: Insects. 2021 May 10;12(5):425. doi: 10.3390/insects12050425 (PMC8150873; doi:10.3390/insects12050425)
Supplement: Supplementary file 1 [file insects-12-00425-s001.zip › insects-1192610-supplementary.pdf]

Supplementary material:

Table S1. Insecticidal activity of different tung meal extracts on termite of *O. formosanus*.

Extracts	Survival rate (% N=20[a])				
	**10[b]**	**5**	**2.5**	**1.25**	**0.625**
**CE**	**0 ± 0**	3.75 ± 4.15	47.5 ± 16.01[bc]	100 ± 0	98.75 ± 2.17
PE	0 ± 0	0 ± 0	85 ± 8.66[a]	97.5 ± 4.33	96.25 ± 4.15
DCM	0 ± 0	1.25 ± 2.17	41.25 ± 20.73[bc]	98.75 ± 2.17	97.5 ± 2.5
EA	0 ± 0	0 ± 0	23.75 ± 10.23[c]	100 ± 0	96.25 ± 2.17
n-Bu	0 ± 0	3.75 ± 6.5	56.25 ± 12.44[b]	100 ± 0	96.25 ± 2.17
H_2O	0 ± 0	13.75 ± 12.44	25 ± 9.35[c]	98.75 ± 2.17	98.75 ± 2.17

N[a], Number of insects tested; [b] Concentrations of test samples, mg/mL; The different letters (a-c) after the mean value represent significant differences at $P < 0.05$ level according to Duncan Test.

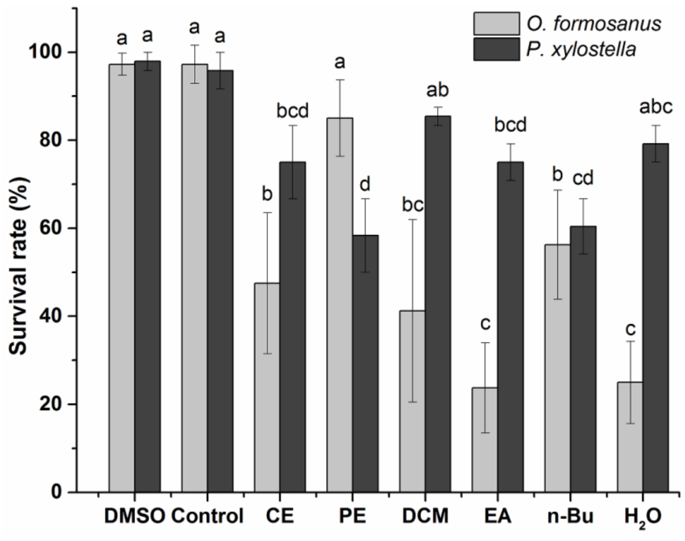

Figure S1. Insecticidal activity of different tung meal extracts on *O. formosanus* and *P. xylostella*. The different letters (a-c) after the mean value represent significant differences at $p < 0.05$ level according to Duncan Test

MS/MS spectrometry fragment profile and fragment ions (relative abundances):

compounds 6:

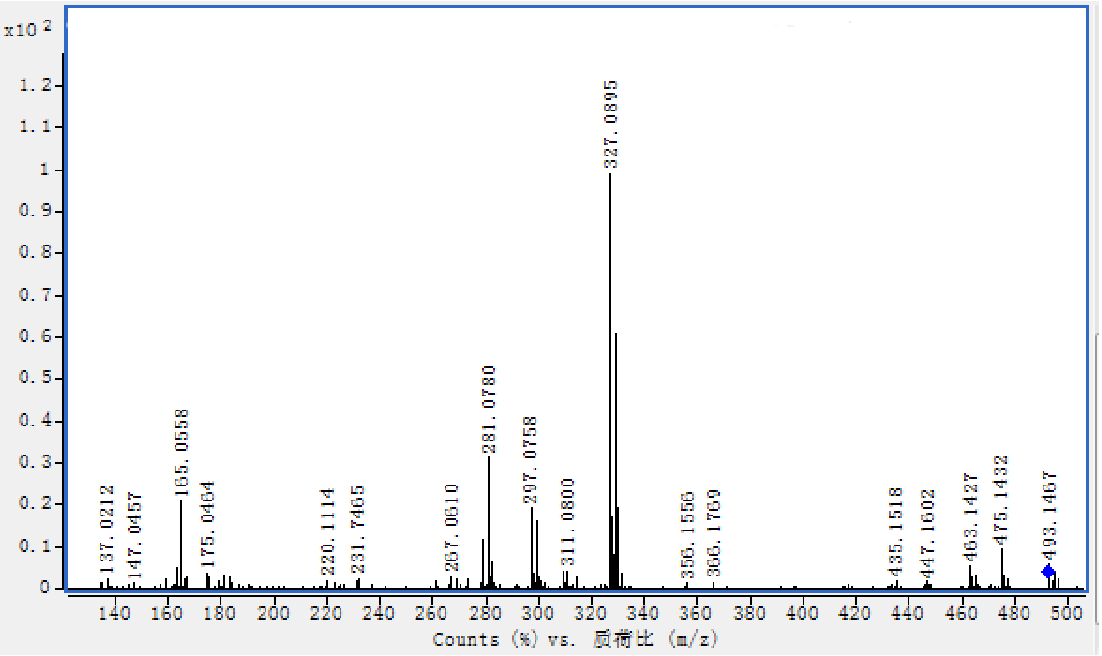

327 (100), 329 (62.09), 281 (31.4), 165 (20.96), 330 (19.47), 297 (19.15), 328 (17.67), 299 (16.63), 279 (12.14), 475 (9.6), 282 (6.14), 493 (5.93), 463 (5.61), 163 (5.18).

Compound 7:

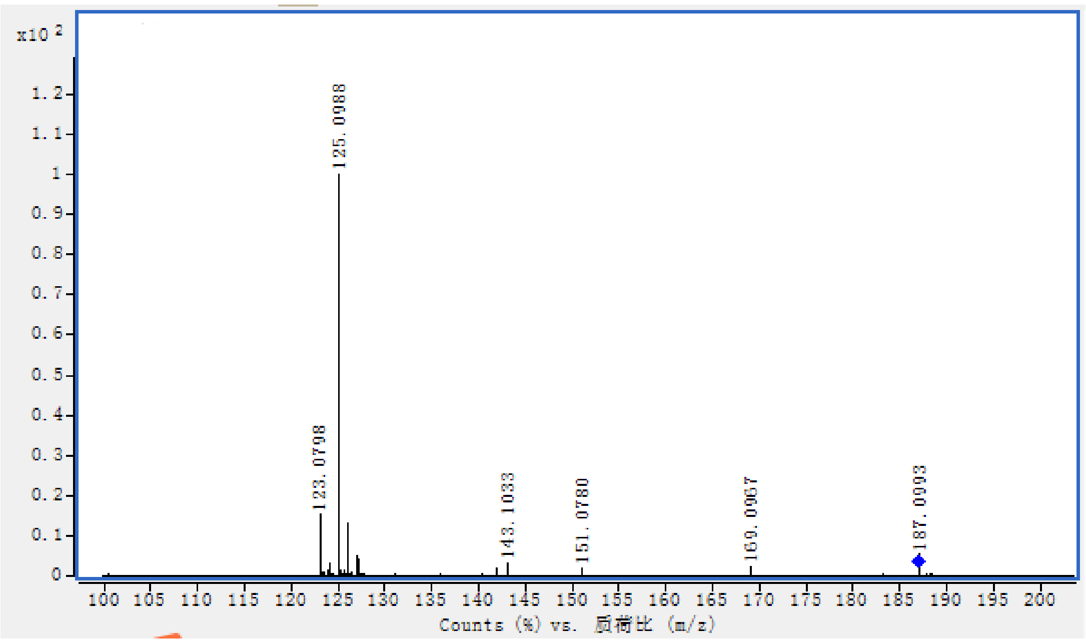

187 (5.25); 169 (2.39); 151 (2.03); 143 (3.23); 142 (1.97); 127 (5.25); 126 (13.59); 126 (1.48); 125 (1.5); 125 (100); 124 (3.37); 123 (15.24).

Compound 9:

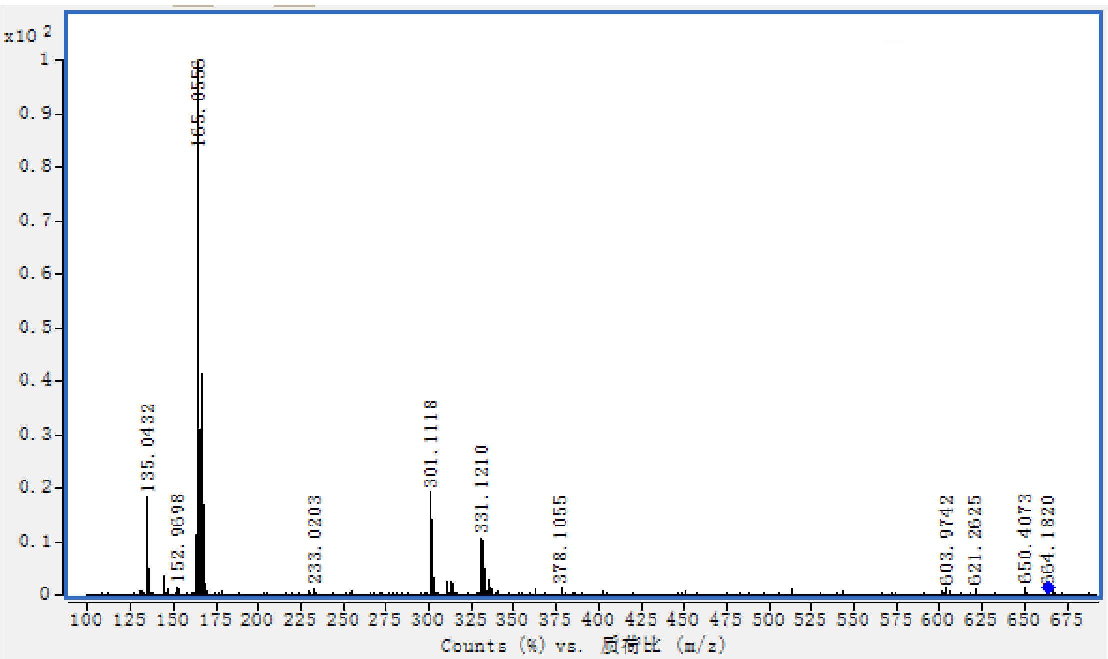

335 (3); 333 (5.21); 332 (10.24); 331 (10.6); 314 (2.25); 313 (2.45); 311 (2.34); 303 (3.15); 302 (14.02); 301 (19.19); 233 (1.23); 169 (2.02); 168 (17.24); 167 (42.92); 166 (30.89); 165 (100); 164 (1.26); 163 (11.64); 153 (1.49); 145 (3.75); 136 (4.99); 135 (18.18).

Compound 10:

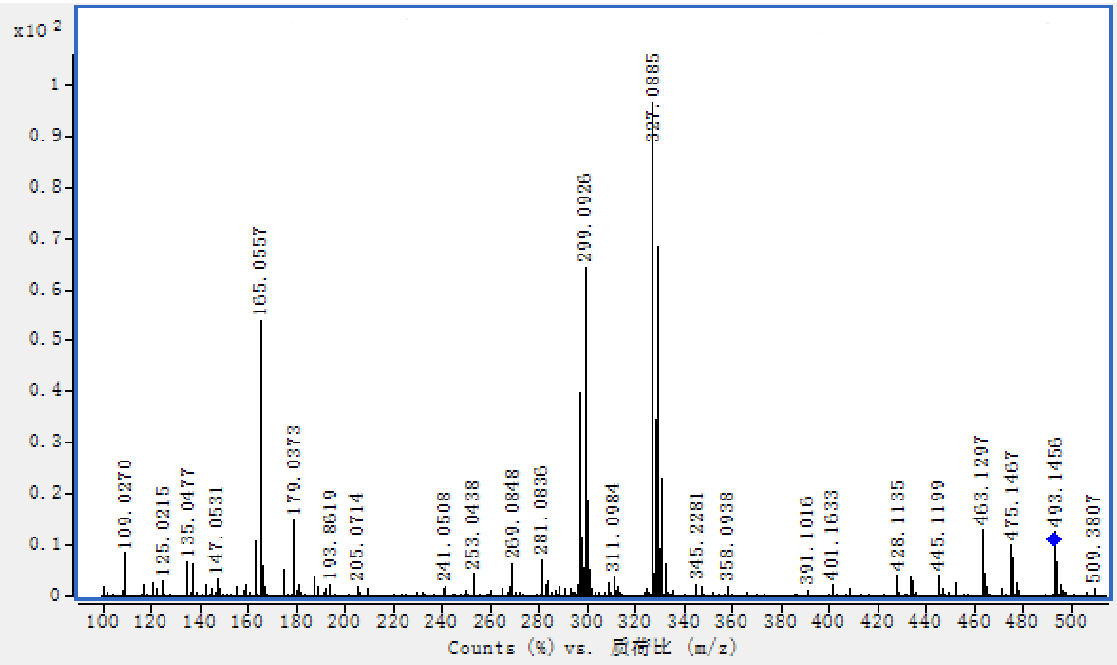

493 (13.05); 476 (7.5); 475 (10.2); 463 (13.27); 331 (23.03); 330 (9.58); 329 (68.73); 328 (34.71); 327 (100); 300 (18.65); 299 (66.11); 298 (11.67); 297 (40.81); 281 (7.26); 269 (6.28); 179 (14.88); 165 (54.09); 163 (11.03); 137 (6.83); 135 (6.88); 109 (8.78).

Compound 13:

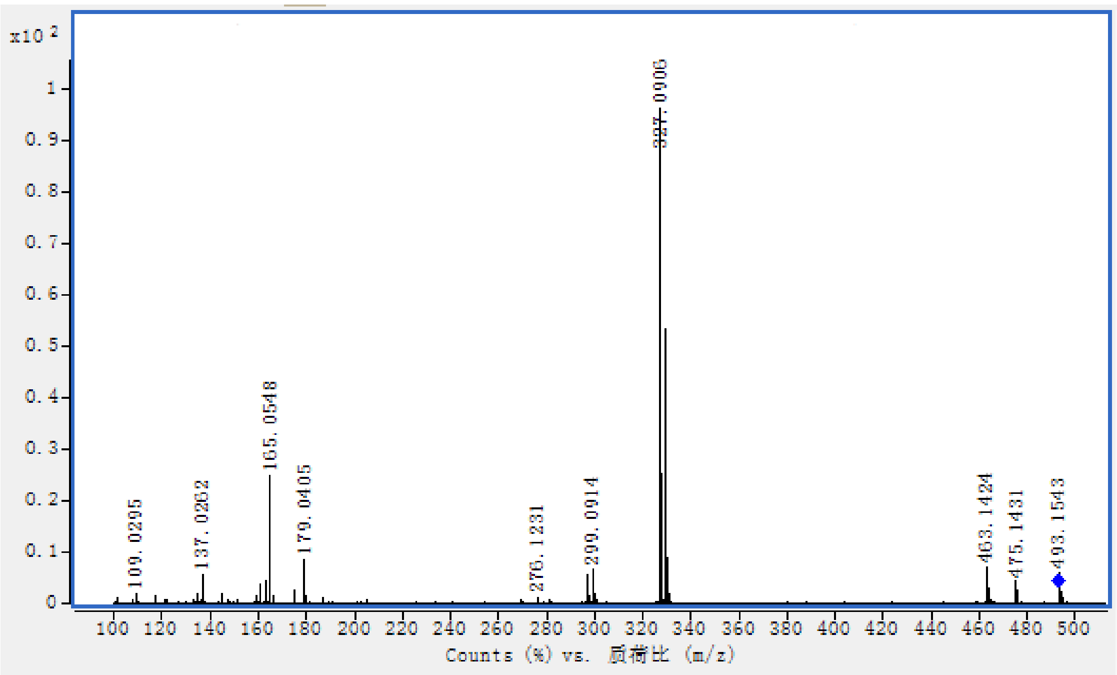

493 (6.16); 476 (2.59); 475 (4.28); 464 (3.1); 463 (7.05); 331 (1.7); 330 (8.91); 329 (53.89); 328 (25.21); 327 (100); 300 (1.87); 299 (6.71); 298 (1.38); 297 (5.66); 276 (1.08); 180 (1.33); 179 (8.91); 175 (2.72); 166 (1.52); 165 (24.79); 163 (4.59); 161 (3.77); 159 (1.45); 145 (1.82).

Compound 14:

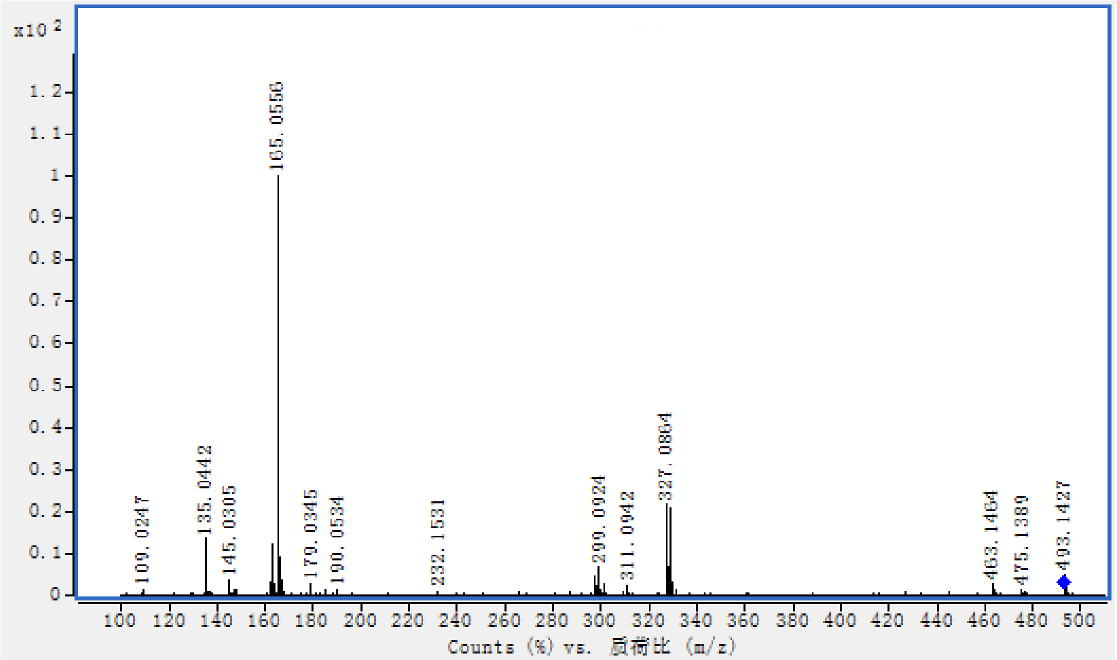

493 (4.89); 464 (1.51); 463 (2.97); 330 (3.49); 329 (21.05); 328 (6.78); 327 (22.17); 311 (2.38); 301 (2.96); 299 (6.66); 298 (2.44); 297 (4.39); 179 (3.01); 167 (3.6); 166 (9.03); 165 (100); 164 (2.83); 163 (12.45); 162 (3.29); 145 (3.58); 135 (13.4).

Compound 15:

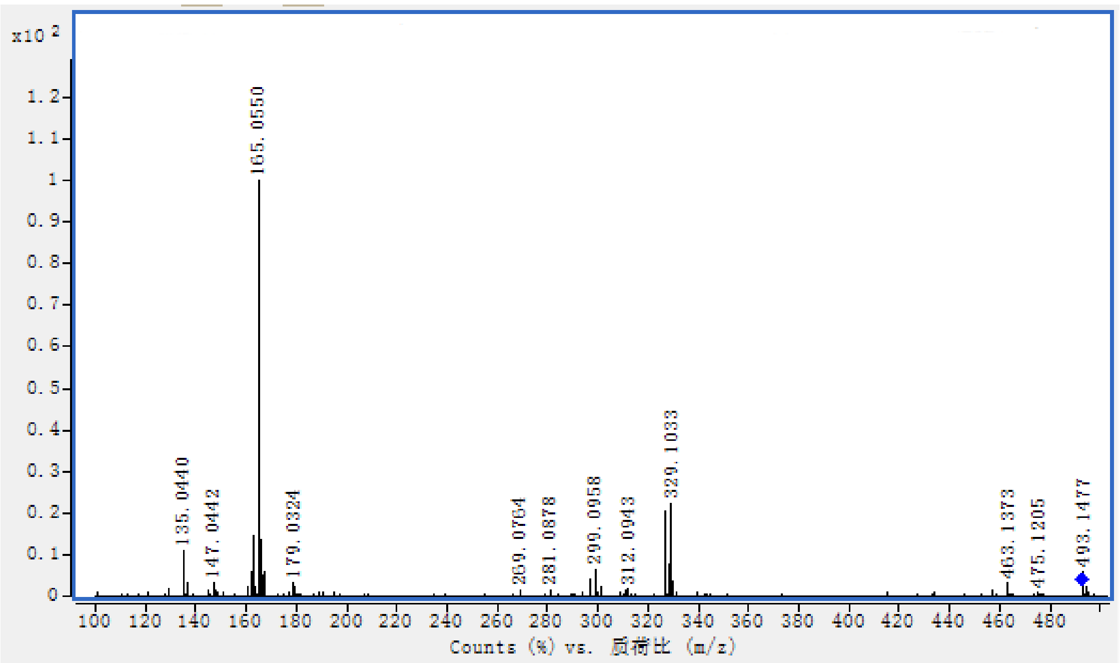

493 (5.81); 463 (3.14); 330 (3.64); 329 (22.3); 328 (7.77); 327 (20.62); 301 (2.36); 299 (6.36); 297 (4.04); 179 (3.48); 167 (5.92); 166 (14.29); 165 (100); 163 (14.39); 162 (5.87); 147 (3.55); 137 (3.39); 135 (10.95).

Compound 16:

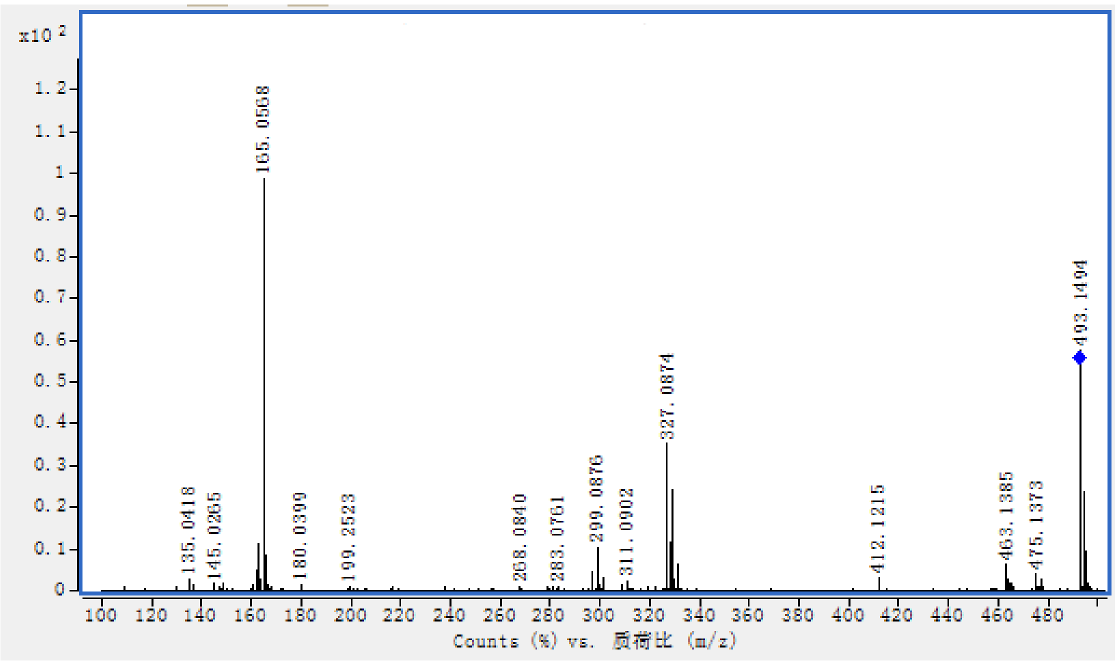

495 (9.18); 494 (23.77); 493 (58.98); 477 (2.66); 475 (4.17); 465 (1.58); 464 (2.75); 463 (6.29); 412 (3.33); 331 (6.2); 330 (2.58); 329 (24.3); 328 (11.76); 327 (36.22); 311 (2.33); 309 (1.52); 301 (3.01); 300 (1.55); 299 (10.42); 297 (4.4); 180 (1.34); 168 (1.16); 167 (1.31); 166 (9.07); 165 (100); 164 (2.8); 163 (11.08); 162 (4.89); 161 (1.36); 149 (1.71); 147 (1.09).

Compound 17:

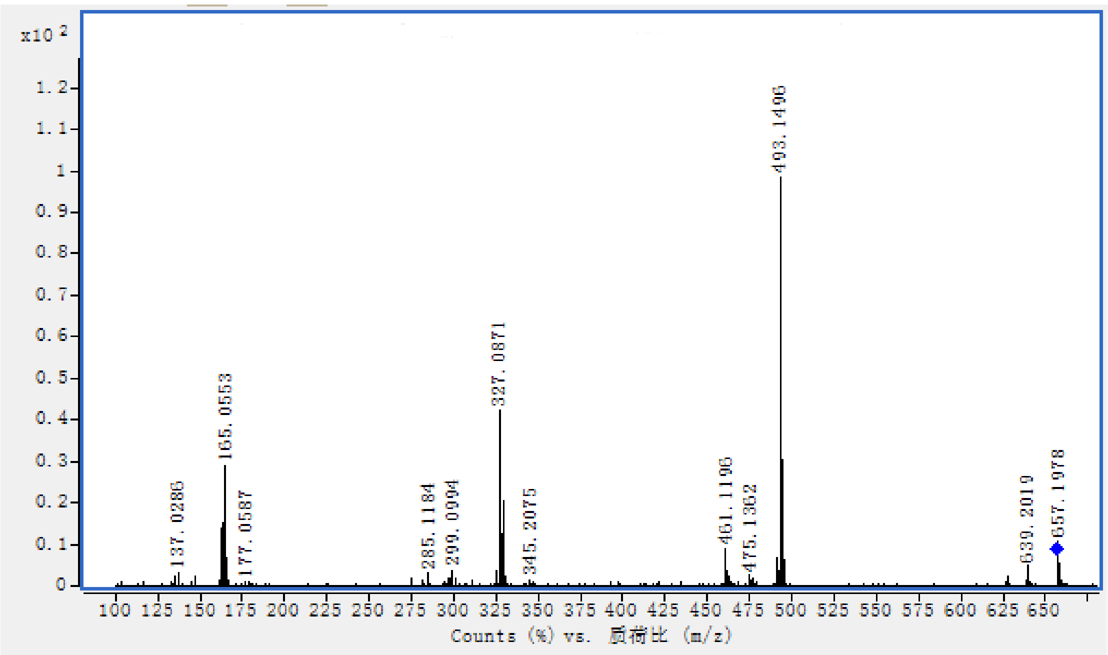

657 (10.89); 640 (2.84); 639 (4.84); 495 (6.46); 494 (30.51); 493 (100); 492 (3.41); 491 (6.51); 475 (2.56); 462 (3.49); 461 (9.31); 330 (4.1); 329 (20.48); 328 (12.65); 327 (43.17); 325 (3.63); 299 (3.72); 285 (3.03); 166 (7); 165 (29.11); 163 (15.31); 147 (2.45); 137 (3.31).

Compound 18:

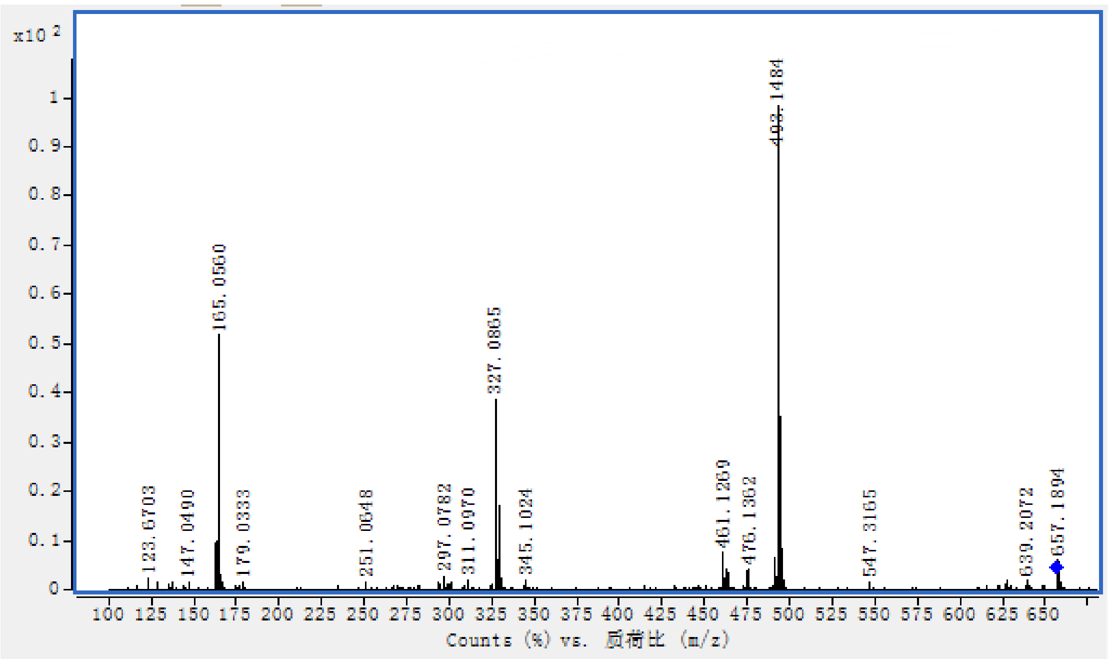

657 (6.29); 639 (2.07); 627 (1.74); 496 (1.96); 495 (8.35); 494 (35.12); 493 (100); 492 (2.66); 491 (6.64); 476 (4.21); 475 (3.91); 464 (3.48); 463 (4.18); 462 (2.22); 461 (7.89); 345 (2); 330 (3.38); 329 (17.29); 328 (6.45); 327 (39.72); 311 (1.96); 297 (2.72); 166 (2.95); 165 (51.71); 163 (10.18); 147 (1.64); 137 (1.79); 129 (1.68); 124 (2.32).

Compound 19:

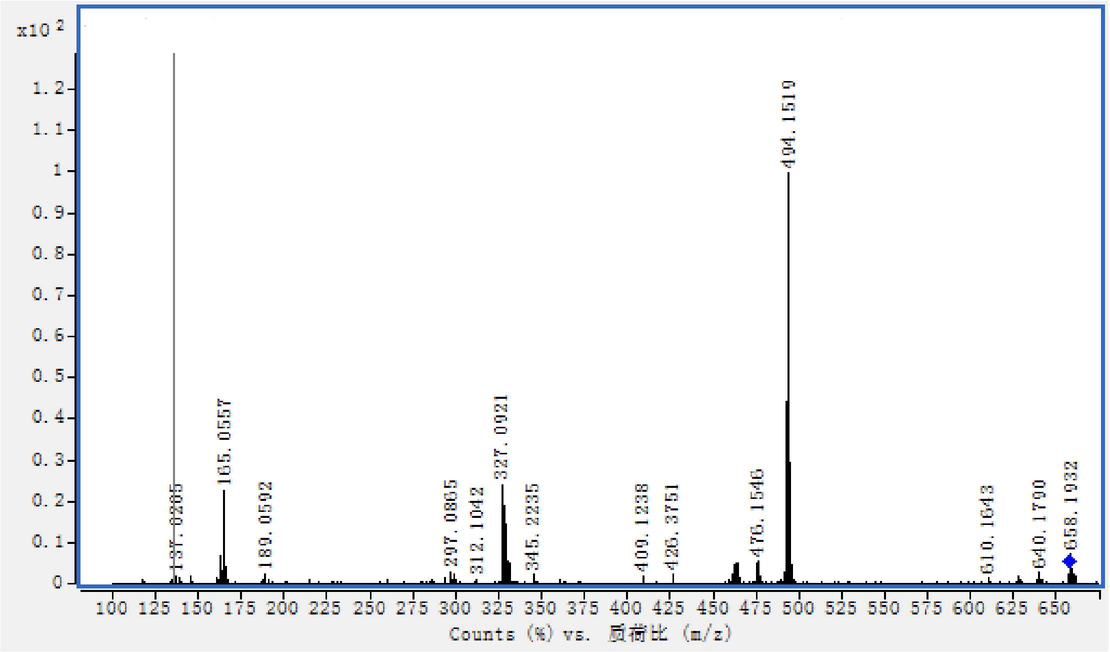

657 (2.43); 495 (29.4); 494 (100); 493 (44.75); 330 (5.61); 329 (14.56); 328 (19.26); 327 (24.03); 165 (22.84); 163 (6.91).
